# Supplementary material for: Surface structure of water from soft X-ray second harmonic generation
Source: Nat Commun. 2025 Nov 26;16:10522. doi: 10.1038/s41467-025-65514-4 (PMC12658203; doi:10.1038/s41467-025-65514-4)
Supplement: Supplementary file 1 — Supplementary Information [file 41467_2025_65514_MOESM1_ESM.pdf]

Supplementary Information to:

Surface structure of water from soft X-ray second harmonic generation

David J. Hoffman[1]*, Shane W. Devlin[2,3,4], Douglas Garratt[1], Sasawat Jamnuch[5,6], Jacob A. Spies[2,7], Bailey R. Nebgen[2,7], Daniel Schacher[4], Alexandria Do[8], Franky Bernal[2,9], Erika J. Riffe[2,9], Kristjan Kunnus[1], Christina Y. Hampton[1], Joseph Duris[1], David Cesar[1], Nicholas Sudar[1], Georgi L. Dakovski[1], Walter S. Drisdell[9], Keith V. Lawler[4], Agostino Marinelli[1], Michael W. Zuerch[2,7], Richard J. Saykally[2,9], Craig P. Schwartz[4]*, Tod A. Pascal[8]*, Jake D. Koralek[1]*

[1] SLAC National Accelerator Laboratory, Menlo Park, CA, United States
[2] Department of Chemistry, University of California, Berkeley, CA, United States
[3] Advanced Light Source, Lawrence Berkeley National Laboratory, Berkeley, CA, United States
[4] Nevada Extreme Conditions Laboratory, University of Nevada, Las Vegas, NV, United States
[5] Theiss Research, La Jolla, CA, 92037, USA
[6] Material Measurement Laboratory, National Institute of Standards and Technology (NIST), Gaithersburg, MD 20899, USA.
[7] Material Science Division, Lawrence Berkeley National Laboratory, Berkeley, CA, United States
[8] Aiiso Yufeng Li Family Department of Chemical and Nano Engineering, University of California, San Diego, La Jolla, CA, USA
[9] Chemical Sciences Division, Lawrence Berkeley National Laboratory, Berkeley, CA, United States

*Corresponding Authors: DJH: djhoff@slac.stanford.edu; CPS: craig.schwartz@unlv.edu; TAP: tpascal@ucsd.edu; JDK: koralek@slac.stanford.edu

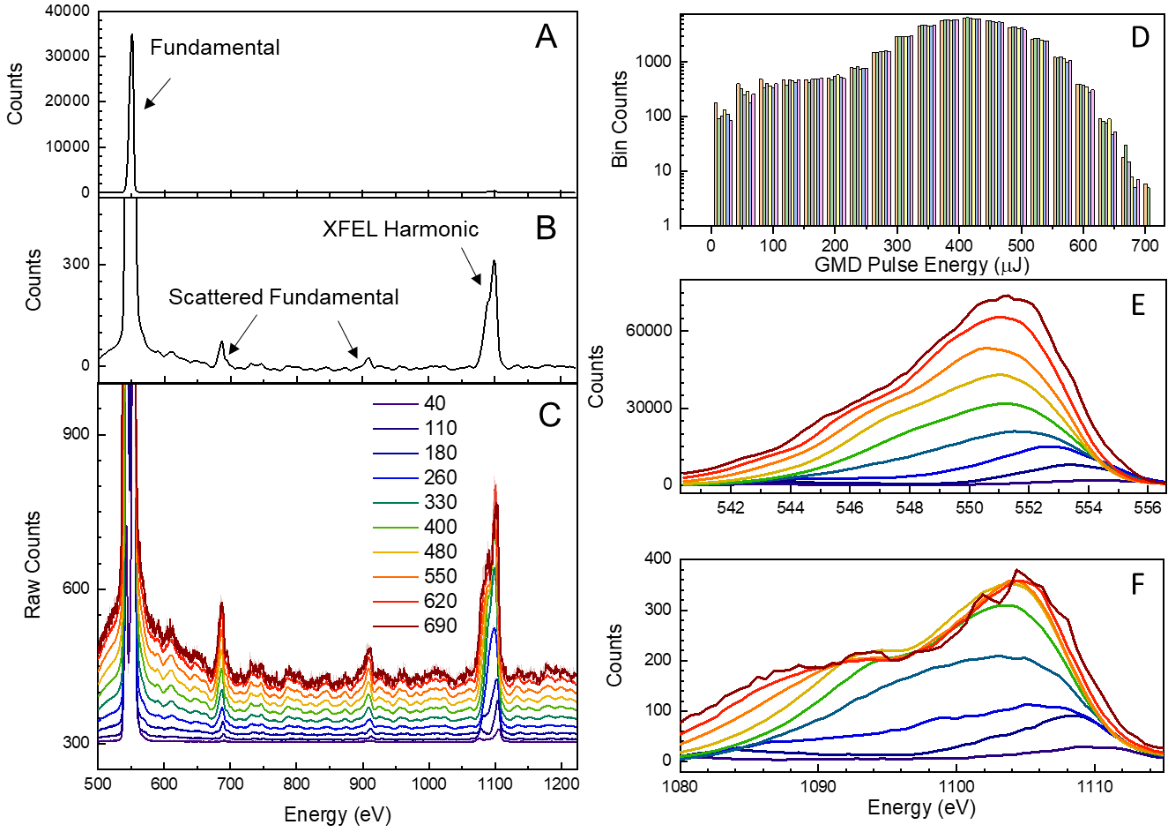

Supplementary Fig. 1. Raw Detector Data. Full detector spectrum with the jet out, showing the relative intensities of the fundamental (**A**) and harmonic (**B**) as well as minor scatter features. **C.** Raw data before background subtraction, binned to gas monitor detector (GMD) pulse energy (μJ). The intensity-dependent scattered light background is visible. **D.** Histogram of GMD pulse energy readings across six separate experimental runs. Baseline-subtracted jet-out fundamental (**E**) and harmonic (**F**) spectra binned to GMD pulse energy.

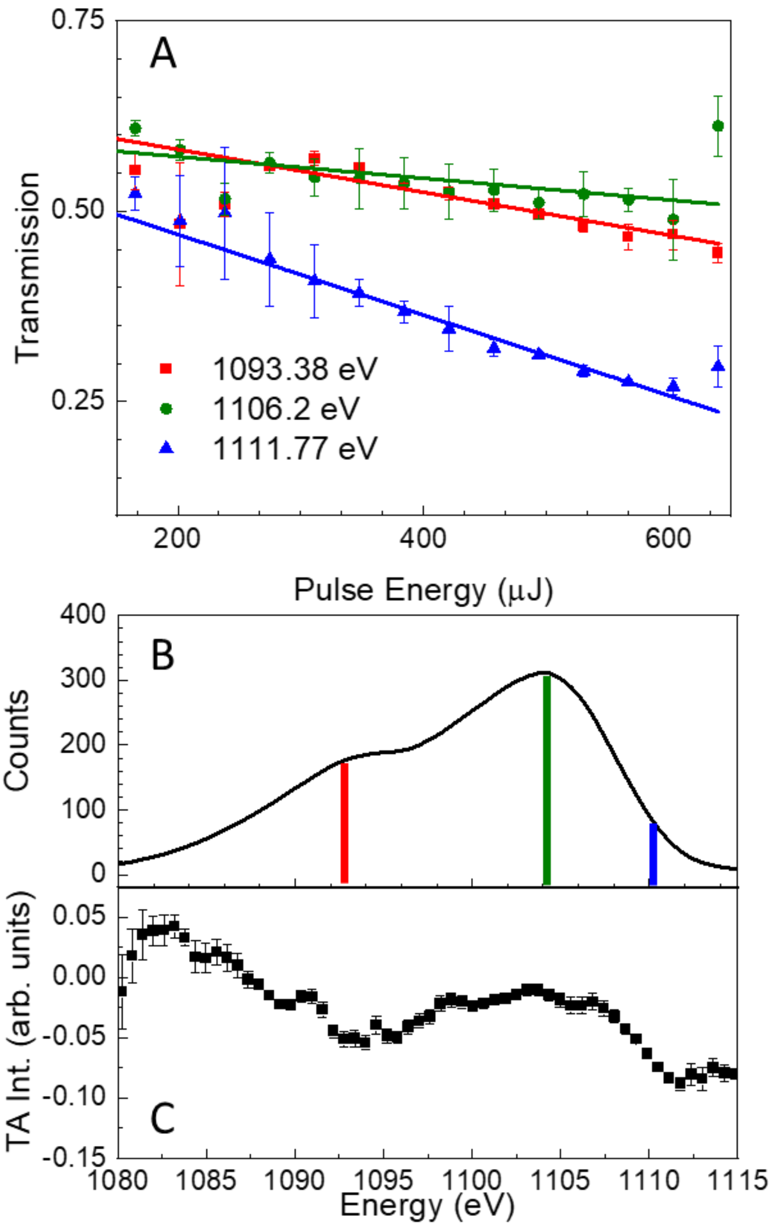

Supplementary Fig. 2. Transient Absorption Intensity and Spectral Dependence. A. Linear fits to the intensity dependent transmission used to estimate the transient absorption (TA) signals. Error bars represent standard error between three measurements. **B.** Corresponding spectral regions of the fits from (A) relative to the jet-out harmonic spectrum. **C.** Estimated magnitude of the TA signal across the spectrum. The non-monotonic behavior indicates potential SHG contributions. Error bars represent standard deviation from the linear fits.

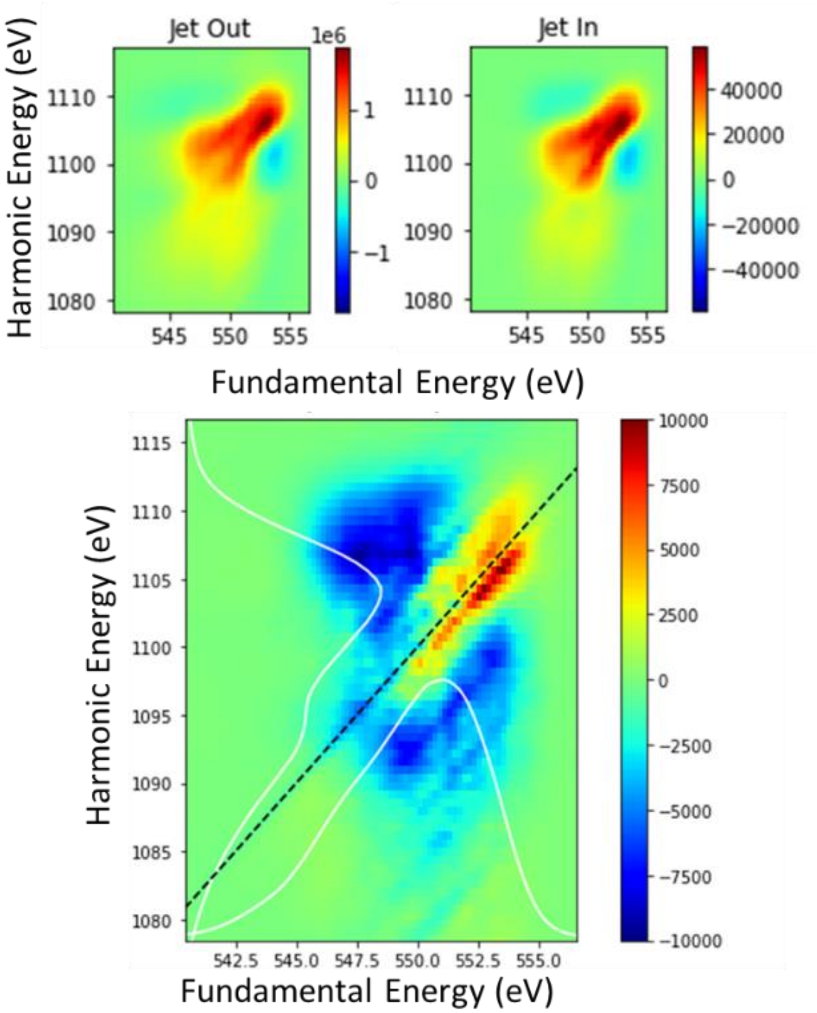

Supplementary Fig. 3. Calculating the Covariance Map. Jet-out (top left) and jet-in (top right) raw covariance maps for a dataset at 550 eV. The jet-out map shows the nontrivial correlations between the machine fundamental and harmonic. The covariance difference map (bottom) is obtained by scaling the jet-out data by the transmission through the jet and subtracting the scaled jet-out from the jet-in data, which follows from the bilinearity of covariance.

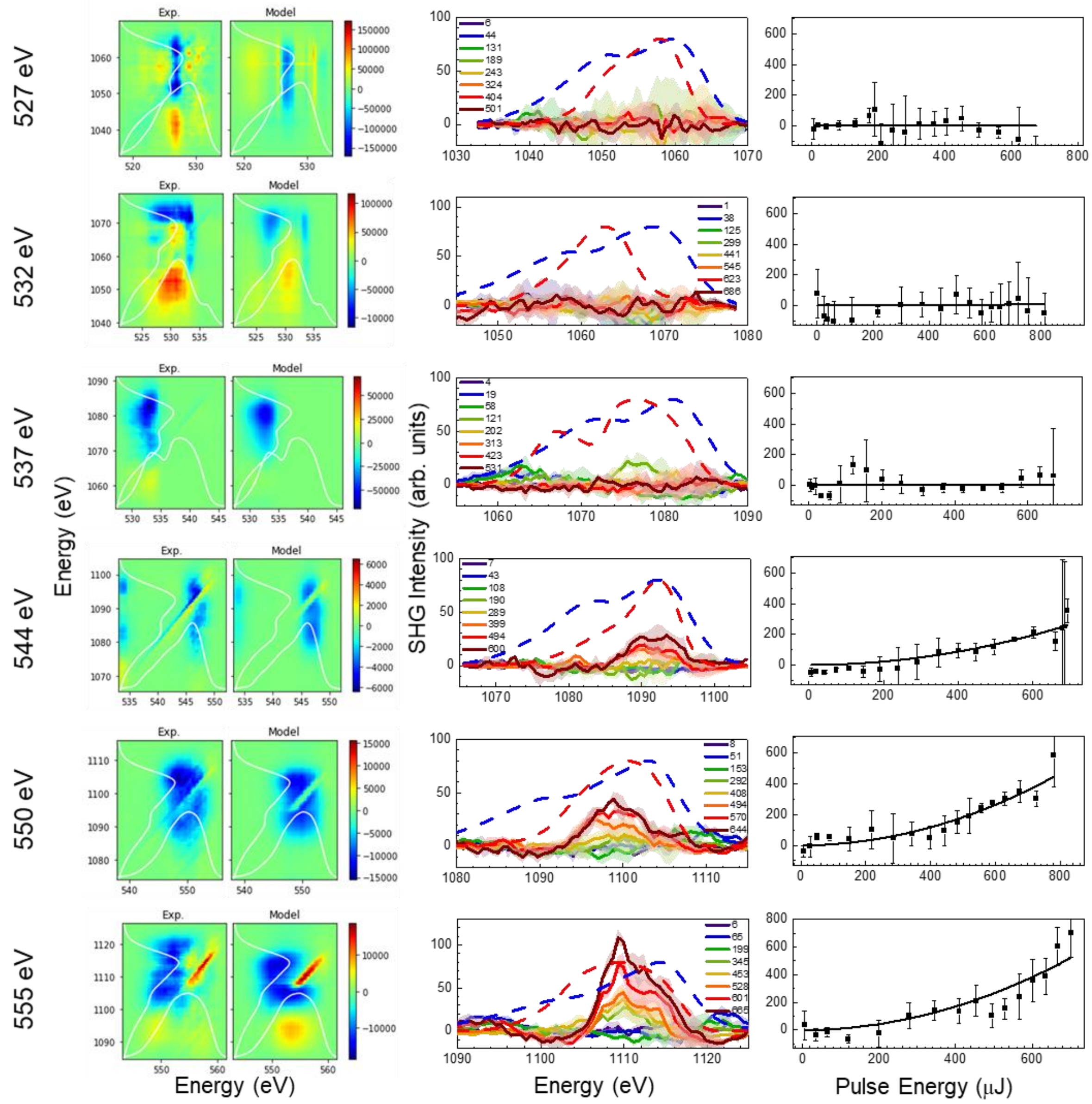

Supplementary Fig. 4. SHG Intensity Dependence at Each Energy Point. Experimental covariance difference maps, modeled covariance difference maps, intensity dependence of difference signal for representative datasets across the oxygen K edge. The colored lines correspond to pulse energy in μJ as in Fig. 3.The dataset shown for 550 eV is from a separate set of runs from the dataset shown in Figs. 2 and 3. Colored shaded regions and error bars represent standard error between three datasets.

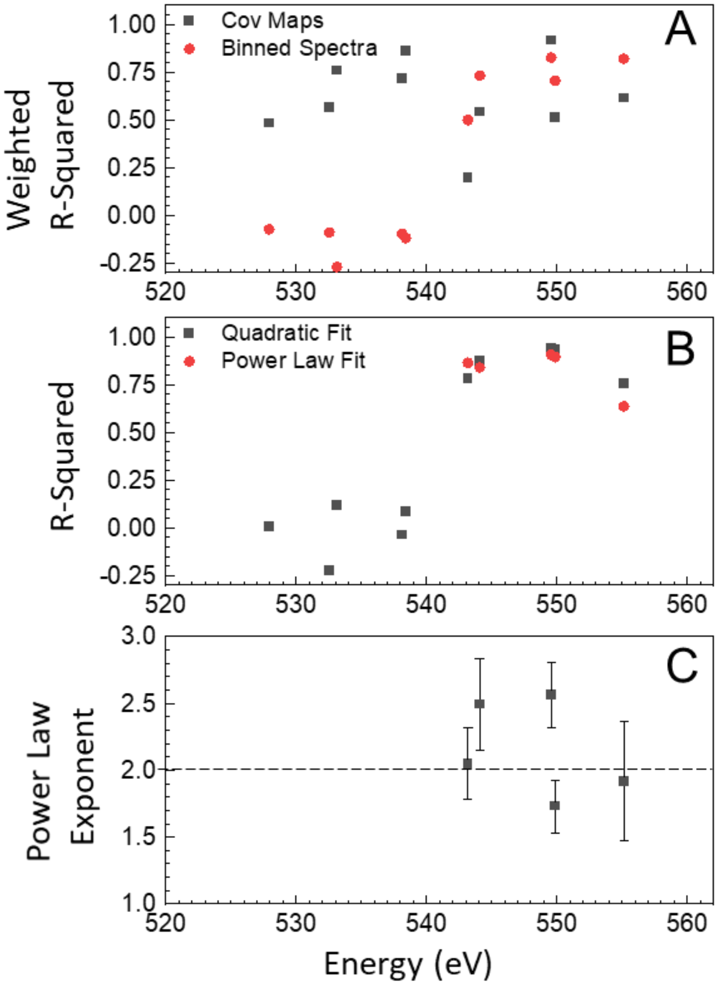

Supplementary Fig. 5. Quality-of-Fit Parameters at Each Energy Point. A. Best fit weighted R-squared values for the covariance map and binned isolated spectra for the fitting procedure for each photon energy. A negative value of the R-squared for the binned spectra indicates an SHG response is disfavored. **B.** R-squared values of the fluence dependent quadratic fits and power law fits. A low R-squared value of the quadratic fit similarly indicates the SHG response is disfavored. **C.** Power law exponents for the fits to data containing candidate SHG signals. The fit exponents fall between 1.5 and 2.5. Error bars represent standard deviation from the nonlinear fits.

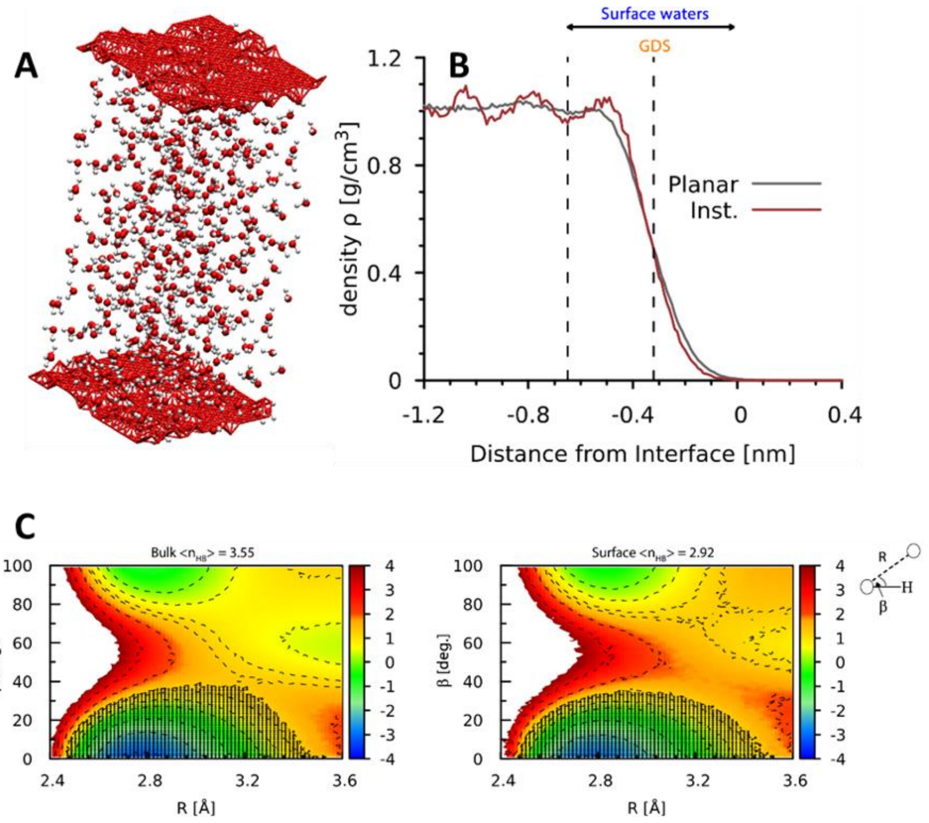

Supplementary Fig. 6. Illustrations of Interface and H-bond Definitions. A. Snapshot of the MB-Pol MD simulation cell used in this study. The computed instantaneous interface is shown by the red mesh. **B**. Mass density profile of water molecules near the interface. Results obtained using the planar (blue) and instantaneous (red) interfaces are compared. Dashed vertical lines denote the interfacial layer, and the Gibbs dividing surface (GDS). **C.** 2D-PMF-based H-bonding in bulk and surface waters, described by the MB-pol potential. The definition of the donor – acceptor distance R and the acceptor-donor-hydrogen angle β are shown by the schematic picture on the right. The contours are in units of kT, and the shaded black regions define the enclosed area passing through the saddle-point at 1.23 kT and bounding the minima at 2.8 Å and 0°. We obtain an average $<n_{HB}>$ of 3.55 HBs in the bulk and 2.92 at the interface

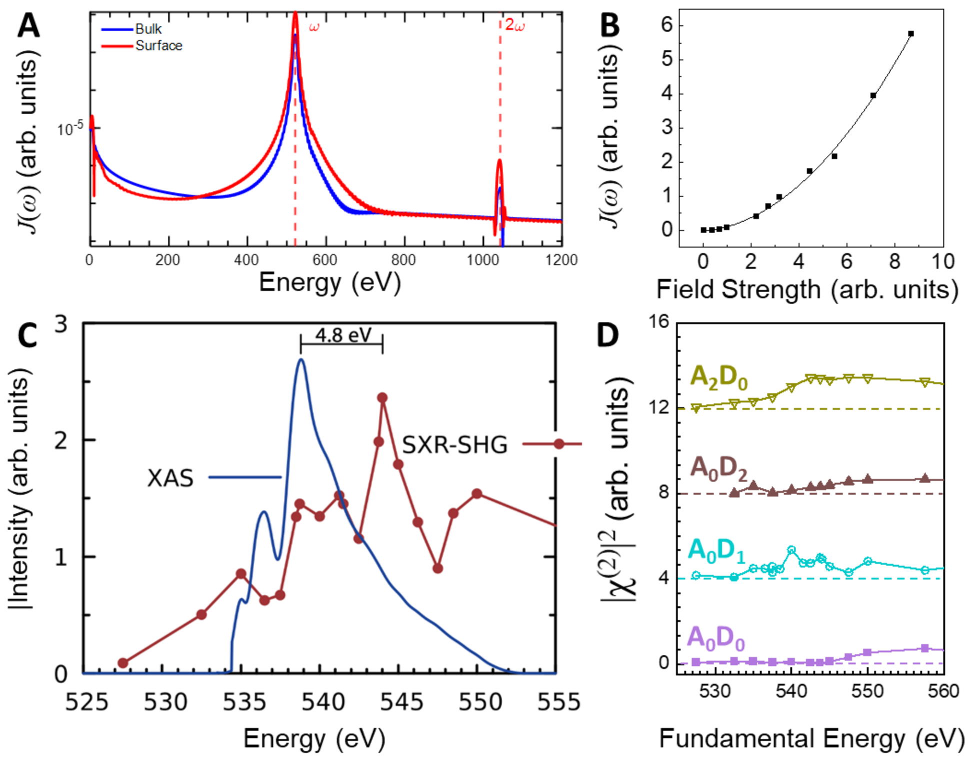

Supplementary Fig. 7. Simulation of the SXSHG Signal. A. Comparison of the total simulated SHG response of water molecules in a slab (2D - red) and bulk (3D - blue) geometry, interacting with a 10^{16} W/cm^2 laser pulse. Although the linear response of systems at the fundamental photon energy ω are comparable, there is an order of magnitude increase in the SHG response of the water surface/slab geometry at 2ω. **B.** Example of the SHG response at a fundamental photon energy of $\hbar\omega = 520.9$ eV. **C**. Comparison of the total simulated spectra of our water slab, showing the bulk, XAS response (blue) compared to the surface-sensitive SXSHG response (red). We find a blue-shift of ~ 5 eV in the main peak of the SXSHG compared to the XAS, consistent with our experiments. This peak is the signature of water molecules accepting 1 hydrogen bond and donating none at the interface **D**. SXSHG spectra for minor H-bonded species not in Fig 4.

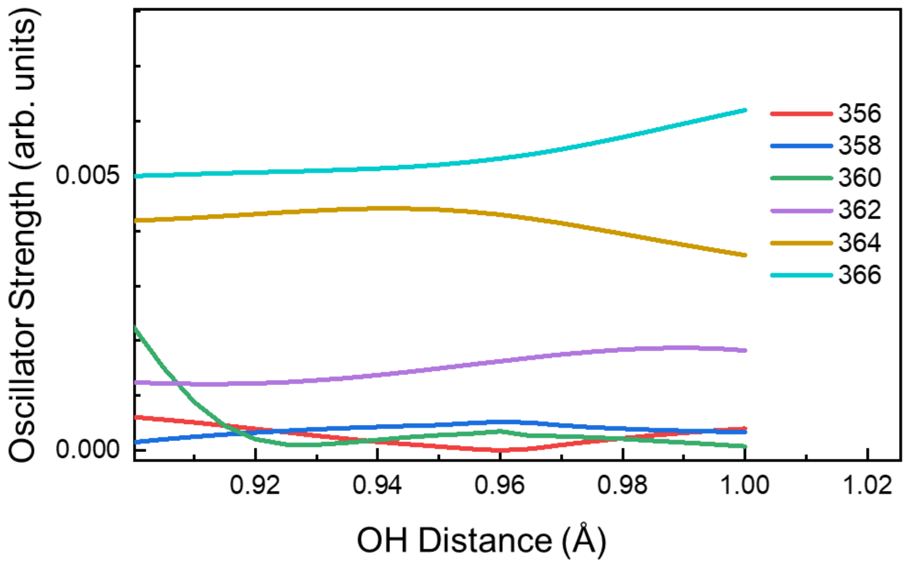

Supplementary Fig. 8. Sensitivity of SXSHG states to molecular geometry. Variations in the strength of the SXSHG states of an isolated water molecule, as a function of stretching one of the OH bonds. The energy of the states at their equilibrium distance are given in Table S2. The states 356, 358 and 360 comprise manifold sx1, are weakly allowed, and are relatively insensitive to OH bond length. The states 362, 364 and 366 comprise manifold sx2 and are intermolecular charge transfer states with stronger oscillator strengths.

Supplementary Table 1: **Hydrogen bond population analysis.** Comparison of the H-bonding properties of bulk and interfacial water, described with the MB-pol(2023) potential and the 2D PMF approach at 298K and 277K

	**$\langle n_{HB} \rangle$**	**%A_0D_0**	**%A_0D_1**	**%A_0D_2**	**%A_1D_0**	**%A_1D_1**	**%A_1D_2**	**%A_2D_0**	**%A_2D_1**	**%A_2D_2**
**298K**										
**Surf**	2.92	0.49	2.18	1.53	3.01	20.33	20.12	1.61	16.54	34.18
**Bulk**	3.55	0.09	0.60	1.18	0.80	7.06	18.72	1.17	12.78	57.61
**277K**										
**Surf**	2.90	0.47	1.06	1.50	3.11	17.92	20.32	1.50	19.23	34.90
**Bulk**	3.67	0.08	0.48	1.16	0.78	5.62	16.30	1.12	11.50	62.92

Supplementary Table 2: States in the sx1/sx2 manifold for an isolated water molecule. The calculated excited states, their oscillator strengths, transition moments and spin state, for a single water molecule. The "state #" is the energy ordering of the state as output by Q-Chem.

**State #**	**Energy**	**Oscillator Strength**	**Transition Moment**			**⟨S^2⟩**
	(eV)		x	y	z	
**355**	1041.0656	3.5496E-04	0	0	-0.0037	0.0000
**356**	1041.1902	0.0000E+00	0	0	0	0.0000
**357**	1041.2199	0.0000E+00	0	0	0	2.0000
**358**	1041.2231	0.0000E+00	0	0	0	2.0000
**359**	1041.3639	2.1215E-04	0	0.0029	0	0.0000
**360**	1041.5178	5.1944E-04	-0.0045	0	0	0.0000
**361**	1049.0922	0.0000E+00	0	0	0	2.0000
**362**	1049.1609	0.0000E+00	0	0	0	2.0000
**363**	1049.9492	4.3045E-03	-0.0129	0	0	0.0000
**364**	1050.0962	1.6316E-03	0	0.008	0	0.0000
**365**	1071.9644	0.0000E+00	0	0	0	2.0000
**366**	1074.1424	5.3374E-03	0	0	0.0142	0.0000
